# Health at work: a critical review of audiovisuals that address workplace exercise

**DOI:** 10.47626/1679-4435-2020-585

**Published:** 2020-12-11

**Authors:** Naiara Carneiro da-Silva

**Affiliations:** 1 Universidade do Estado da Bahia, Licenciatura em Educação Física - Campus IV - Jacobina (BA), Brazil

**Keywords:** work, sickness, workplace exercise

## Abstract

Work has an important role in people’s lives: in addition to providing means of living, it allows people to feel useful, productive, and valued. However, when performed in inadequate conditions, it can damage the worker’s health and cause diseases that result in physical and occupational inactivity. The aim of the present study is to critically analyze the content of videos addressing workplace exercise. Our exploratory and quantitative review was performed on the YouTube video platform in April 2019, using the descriptor “ginástica laboral” (in English, workplace exercise). We selected 104 Portuguese language videos according to their number of views. Out of these videos, only 11 provided complete information regarding workplace exercise; these were interviews with physical education and physiotherapy professionals that highlighted the need for and benefits of physical activity in maintaining worker health. In addition, these videos illustrated the correct execution of exercises, emphasizing the importance of company investment in workplace exercise for the health and well-being of their employees. This study demonstrates that, altogether, the quality of videos on this subject available on YouTube is low. Their publication is not automatically subjected to classic scientific review nor it is guaranteed by an academic expert, which results in variable quality and inaccurate or incomplete information that can easily propagate through this virtual platform and be falsely perceived as correct.

## INTRODUCTION

The industrial era represented the beginning of mass production and an increasing worker specialization aimed at improving product quality, increasing production rates, and reducing costs. This specialization led workers to execute specific tasks within companies, performing repetitive movements that were associated to excessive force and ultimately led many workers to experience different types of pain.^1^

Work has an important role in people’s lives, providing means of living and allowing them to feel useful, productive, and valued.^2^ However, when performed in inadequate conditions, it can be harmful to one’s health and cause illnesses that lead to physical and occupational inactivity. Among health problems caused by work, notable examples include repetitive strain injury (RSI) and work-related musculoskeletal disorders (WMSD), as well as psychological illnesses related to stress. Considering these issues, companies of different sectors are investing in activities that reduce occupational problems caused by excessive work, such as workplace exercise.^3^

Workplace exercise includes specific exercises for promoting stretching and muscle strengthening, as well as motor coordination and relaxation; these are performed in different sectors or departments of the company with the main objective of preventing and reducing cases of RSI, WMSD, and stress.^4^ These programs can have broad objectives that include improvements in self-knowledge, self-esteem, or interpersonal relationships within the workforce. Altogether, it is important to stimulate studies that aim to identify prevention strategies against adverse events related to health care assistance.

Meanwhile, we note the importance of YouTube, which currently represents a global source of information particularly regarding health problems. Most of the available videos are not subjected to specialist review and are not, therefore, properly scientific. Hence, it is of utmost importance to verify the quality of the available material.^5^ Considering the unquestionable importance of internet videos in the current era, videos that approach health care aspects could result in erroneous actions and/or assumptions and compromise the patient’s health and security.^6^

Within this perspective, the aim of this study was to evaluate the content of YouTube videos regarding workplace exercise, acknowledging their importance in health promotion as sources of information and caution regarding worker health. Therefore, we have performed a critical review of these videos, verifying the quality of their content and correlating it with the literature.

### HISTORY OF YOUTUBE

The internet currently represents an extremely important source of information that is unquestionably part of peoples’ lives. The growth of services that allow media distribution and sharing provided people access to various products and has transformed people into multi-media beings. The internet processes virtual information and transforms it into our reality, constituting a society that functions as a network.^7^

Before the appearance of YouTube in 2005, internet users had only a few simple options when it came to uploading their videos. With a user-friendly interface, YouTube allowed any user to upload videos and potentially reach millions of people in a few minutes. The great variety of subjects available in its videos has turned the platform into one of the most important aspects of internet culture.

One can watch and share from home-made videos to TV shows, interviews, critics, and denunciations anytime and anywhere. The diversification of the interfaces through which users can interact and produce content is one of the characteristics associated to the so-called “web 2.0.” Therefore, any one individual can access YouTube to obtain information on various subjects, including health care.^8^

YouTube is classified by Google as a platform of content distribution. It thus consists in an online database of audiovisual products that allows users to upload, share, produce, and publish, in a digital format, home-made or professional videos; it is the most popular website of this type and consequently a valuable tool in contemporary society.^9^

## METHODOLOGY

We performed an exploratory review, with a quantitative approach, using the YouTube video sharing website. Our research was performed in April 2019 using the descriptor “ginástica laboral” (in English, workplace exercise), according to the Health Sciences Descriptors (DeCS).

We made the search using the mentioned descriptor and then used the website’s “view count” filter to choose videos with at least 1000 views. Aiming to obtain a representative sample even with only one descriptor, we considered 104 videos in our initial sample.

After this initial search, the videos were individually evaluated and our inclusion criteria considered videos with direct references to workplace exercise, practical demonstrations, and Portuguese language interviews. The videos that did not correspond to our criteria, in addition to lectures and online classes, were excluded. For evaluating the videos, we elaborated a closed-question research instrument.

After video selection according to inclusion and exclusion criteria, our sample was analyzed according to: muscle groups in question; length of hold for each stretching exercise; benefits of workplace exercise; use of group activities and equipment; inclusion of warmup and relaxation phases; use of music; duration of the videos; educational background of the person responsible for the video; year of upload; and view and like/dislike count.

These aspects were evaluated through descriptive statistics. Our study did not require approval by an ethics committee since this research did not work directly with human beings and used publicly available material.

## RESULTS

**Figure 1:**
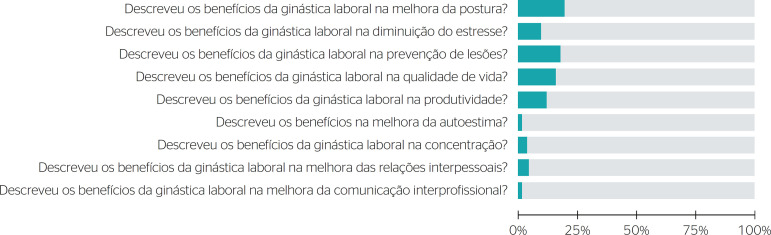
Benefits of workplace exercise.

Among the 104 included videos, the most addressed subjects were: benefits of workplace exercise in posture improvement (20.8%), stress reduction (9%), injury prevention (15%), and in quality of life (15%) and productivity (12%) (Figure 1).

**Figure 2:**
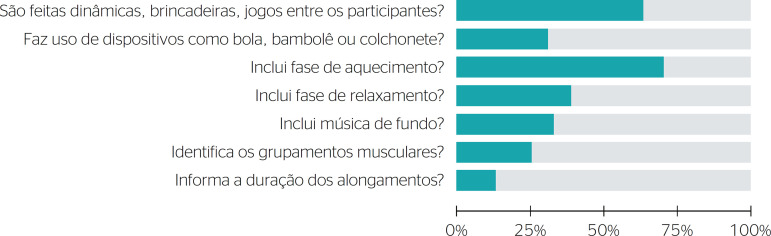
Practical demonstrations

Regarding the videos that showed practical demonstrations, the warmup phase was the most addressed aspect (71.6%); on the other hand, the length of stretch exercises was only approached by 13 videos (13.5%). Group activities such as games were the second most performed aspect and were present in 65% of the videos. Only 25% of the videos identified muscular groups during exercise execution (Figure 2).

Table 1 presents the mean duration of videos and their number of views, likes, and dislikes.

**Table 1:** Relative data

	Views		Evaluation			Ratio	Duration
	Mean	SD	Likes	SD Dislikes	SD	Likes/dislikes	Mean (seconds)
Audiovisuais	15679	37.80	2124	194.77 104	19.64	20.42	155

SD: standard deviation.

## DISCUSSION

The analysis of audiovisual content using YouTube videos is a recent activity, since the platform started its activities in 2005. It is currently the second most visited website worldwide and has over 1 million users, which comprises almost one-third of all internet users.^10^ Owing to its ease of use and visibility, YouTube has been used^11^ in virtually all kinds of communication, which is why it has become popular among patients and health care professionals as a source of medical information and as an educational tool.

This study has some limitations. Firstly, we used YouTube as our only source website, and there are other available tools for online video sharing. Secondly, we only selected Portuguese language videos, which restricted our sample selection. One of the main inclusion criteria was the number of views, which reflects a video’s popularity in a certain moment. This way, some videos with high numbers of views were excluded since they were in other languages.

One final limitation is inherent to any research covering YouTube content: We only covered one moment in time. This website is a dynamic platform in constant growth that consequently changes rankings and view numbers and receives new videos at a high pace.

The included videos had been uploaded from 2007 to 2019 and were collectively watched 15 679 times. An important aspect of this sample was that none of these videos actively mentioned the importance of workplace exercise in worker health, the effects of these exercises, and their efficacy in promoting health and wellbeing in the workplace.

The videos highlighted some of the benefits of workplace exercise such as posture improvement, stress reduction, injury prevention, and increases in quality of life and productivity. Benefits to self-esteem and interprofessional communication were not mentioned. Most of them (71.6%) mentioned stretching exercises in the warmup phase and the use of group activities (65%), despite not informing the duration of the activity or how long each stretch should be held.

According to the literature, workplace exercise comprises physical activity performed at the worksite in 5-, 10-, or 15-minute sessions, aiming to prevent RSI and WMSD and to reduce stress through stretching and relaxing exercises.^12^ Workplace exercise helps in promoting worker health and should provide individual changes such as improvements in pain, posture, and self-esteem, stress reduction, a better quality of life and both personal and collective advantages through benefits to the work environment.^13^

According to Silva,^14^ workplace exercise promotes the health of workers and reduces work-related illnesses and injuries due to repetitive movements. Lima et al.^15^ reported that these exercises aim to relax or tone the body structures that are used the most in the work environment, in addition to activating those that are less required. Finally, according to Freitas et al.^16^ and Machado Junior et al.,^17^ workplace exercise also includes measures that combat physical and emotional disorders, in order to primarily prevent potential diseases caused by repetitive and monotonous work.

Workplace exercise provides benefits both to the workers and the company. In addition to preventing RSI and WMSD, it has more fast and direct outcomes such as improvements in interpersonal relationships and body pain.^18^

Soares et al.^19^ state that workplace exercise programs have better results and adherence when constantly supervised by specialized professionals such as physical educators or physiotherapists. Out of the analyzed videos, only 19% were presented by physical educators and 5% displayed physiotherapists, while 68% did not specify the educational background of the instructor.

Moreover, only 11 out of 104 videos brought complete information regarding workplace exercise - mostly interviews with physical education and physiotherapy professionals who explained the need for workplace exercise and its benefits in worker health. In addition, the interviewees also demonstrated the correct execution of the exercises and highlighted the importance of company investment in workplace exercise for the health and wellbeing of employees.

## CONCLUSIONS

Altogether, the studied videos did not approach all benefits of workplace exercise in worker health, which compromised the quality of the shared material. Social media and video sharing websites such as YouTube have become a part of our daily life and the number of videos on health-related subjects increases daily. YouTube is currently second only to a doctor’s advice as the main source of information regarding health-related topics.^20^

Our study indicates that the overall quality of these videos is low. Video uploads to the YouTube platform are not automatically subjected to classic scientific review or to academic expert inspection, which results in variable content quality and the dissemination of incorrect or incomplete information that can easily spread through this virtual platform and perceived as correct. This corroborates the hypothesis that YouTube material should be treated carefully and be subject to critical evaluations of precision and reliability.
